# CRISPR Interference to Inhibit Oncogenes for Cancer Therapy

**DOI:** 10.3390/ijms27083564

**Published:** 2026-04-16

**Authors:** Bin Guo

**Affiliations:** Department of Pharmacological and Pharmaceutical Sciences, University of Houston, Houston, TX 77204, USA; bguo@central.uh.edu

**Keywords:** CRISPR interference, CRISPR-Cas, CRISPRi, gene silencing, oncogenes, cancer therapy, gene delivery

## Abstract

CRISPR interference (CRISPRi), a programmable transcriptional repression technology derived from nuclease-deficient CRISPR-Cas systems, has emerged as a powerful method for selectively inhibiting oncogene expression without altering the genomic DNA. This feature offers a major advantage over other oncogene targeting technologies such as CRISPR-mediated gene knockout, mRNA inhibition by siRNA or miRNA, or small-molecule inhibitors of the proteins encoded by the oncogenes, especially in cancers driven by transcriptional dysregulation or otherwise undruggable oncogenes. Here, I present a comprehensive review of CRISPRi mechanisms, delivery strategies, and preclinical applications in oncology (including advances in targeting core oncogenic drivers like MYC and KRAS). The advantages of CRISPRi as well as in vivo validation of CRISPRi-mediated tumor suppression are discussed. Finally, I outline translational challenges and future directions for incorporating CRISPRi into precision cancer therapies. The accumulated evidence suggests that CRISPRi could become a cornerstone for next-generation gene-regulatory therapeutics.

## 1. Introduction

Cancer progression is fundamentally driven by gain-of-function mutations or overexpression of oncogenes or loss-of-function mutations in tumor suppressor genes [1,2,3]. Classic examples include KRAS [4,5], which when mutated keeps cell division signals permanently turned on, and MYC, a transcription factor that, when overexpressed, drives rapid metabolic reprogramming and uncontrolled proliferation in lymphomas and carcinomas [6,7,8]. Conversely, tumor suppressors like TP53 (the guardian of the genome) or RB1 act as the brakes [9,10]. Their inactivation removes the cell’s ability to repair DNA or undergo apoptosis, allowing damaged cells to survive and expand.

The pharmacological arsenal against the oncogene drivers has relied on small molecule inhibitors and monoclonal antibodies. These have seen remarkable success; for instance, imatinib revolutionized the treatment of chronic myeloid leukemia by targeting the BCR-ABL fusion protein [11,12], and trastuzumab significantly improved outcomes in HER2-positive breast cancer [13,14]. However, these protein-level interventions face two systemic hurdles. First is the inevitable development of acquired drug resistance, where tumors evolve secondary mutations (such as the T790M gatekeeper mutation in EGFR) that render inhibitors ineffective [15,16]. Second is the persistent challenge of “undruggable” proteins. Many high-priority targets, such as the aforementioned KRAS mutants or the MYC transcription factor, lack deep hydrophobic binding pockets suitable for small molecules, or are intracellular, making them inaccessible to large monoclonal antibodies [17,18].

To overcome these limitations, gene silencing technologies have emerged as a transformative alternative. By shifting the focus from the finished protein to the nucleic acid level, strategies like RNA interference (RNAi) [19,20,21] and antisense oligonucleotides (ASOs) [22,23] aim to degrade the messenger RNA (mRNA) before it is translated. This upstream intervention offers a programmable platform to silence virtually any gene, including those previously deemed undruggable, providing a pathway to halt disease progression by preventing the production of oncogenic proteins at their source. The foundation of gene silencing was laid by small interfering RNAs (siRNAs) [24,25] or short hairpin RNAs (shRNAs) [26,27] to induce the post-transcriptional degradation of target mRNA. Mechanistically, siRNAs are incorporated into the RNA-induced silencing complex (RISC), where the passenger strand is discarded, and the guide strand directs the enzyme Argonaute 2 (AGO2) to a perfectly complementary target mRNA [28]. This leads to site-specific endonucleolytic cleavage and subsequent degradation, effectively turning off protein production. However, clinical translation of RNAi faces significant hurdles, including transient effects and off-target activity, where the siRNA binds with partial complementarity to unintended mRNAs [29]. Unlike siRNAs, microRNAs (miRNAs) typically bind to the 3′ UTR of multiple transcripts to cause translational repression or mRNA decay [30,31], making them potent but complex therapeutic targets for diseases like hepatitis C (Miravirsen) [32]. In addition, ASOs have gained significant clinical momentum. Unlike double-stranded siRNAs, ASOs are single-stranded synthetic polymers that bind to target RNA and trigger degradation via RNase H1 or modulate splicing [33]. This technology has yielded FDA-approved breakthroughs such as Nusinersen (Spinraza) [34] for spinal muscular atrophy and Eteplirsen for Duchenne muscular dystrophy [35].

Both RNAi and ASOs are subjected to a potential resistance mechanism that is also associated with the small molecule inhibitors of proteins [36]. As the mRNAs or proteins encoded by the oncogene are inhibited by RNAi/ASO or small molecule drugs, the oncogenes can continue to express more mRNAs and proteins to overcome the inhibition. As a result, drug resistance will inevitably develop. To overcome this problem, a novel gene silencing strategy based on the Clustered Regularly Interspaced Short Palindromic Repeats (CRISPR)-Cas system has been developed in recent years [37,38,39]. This review will discuss the emergence of CRISPR Interference (CRISPRi) as an effective platform for targeted oncogene inhibition. CRISPRi leverages the precision of the CRISPR/Cas system to repress gene expression directly at the DNA level, offering a novel and potentially more robust strategy for cancer therapy.

## 2. The Molecular Mechanisms of CRISPR Interference

### 2.1. The Dead Cas9 (dCas9) Core

**Figure 1:**
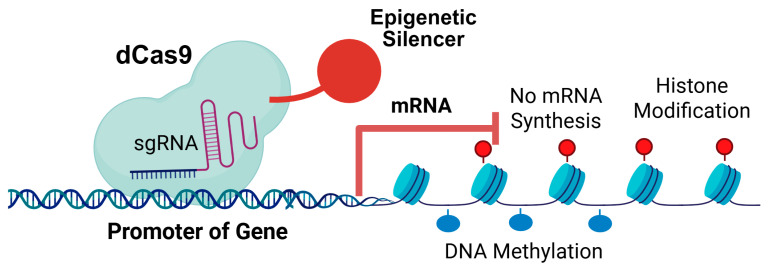
The mechanism of action of CRISPRi. The dCas9 protein is fused with an epigenetic silencer protein (such as KRAB, HDAC1, or DNMT3a). The fusion protein will be guided to the promoter of an oncogene by sgRNA. Modification of histones or methylation of the cytosines at the promoter will inhibit oncogene expression. Inhibition of mRNA synthesis is indicated by the red T line. Created in BioRender. Guo, B. (2026) https://BioRender.com/xj4cbqp.

CRISPRi is a modification of the canonical CRISPR/Cas9 system [40], which originally evolved as an adaptive immune mechanism in bacteria. The core component is catalytically inactive Cas9 (dCas9), engineered by introducing specific point mutations (D10A in the RuvC domain and H840A in the HNH domain) of Streptococcus pyogenes Cas9 [41]. These mutations abolish the enzyme’s endonuclease activity, rendering it incapable of creating double-strand breaks (DSBs). Despite this loss of the DNA cutting activity, dCas9 retains its high-affinity and precision binding to the DNA, guided to a genomic locus by a single guide RNA (sgRNA) and a required Protospacer Adjacent Motif (PAM, 5′-NGG-3′, where “N” is any nucleotide followed by two guanine bases) [41,42]. The binding mechanism is a multi-step process [43]. First, the dCas9 protein must form a ribonucleoprotein (RNP) complex with a sgRNA. This complex scans the genome for a specific PAM. Once the PAM is recognized, the dCas9 protein triggers the local unwinding of the DNA duplex. This allows the seed region of the sgRNA to invade the helix and find complementarity. If the sequence matches, a stable R-loop is formed, locking the dCas9 firmly onto the target site [44]. In CRISPRi, this physical occupancy creates transcriptional interference. By binding specifically to the promoter or the elongation path of a gene, the dCas9 complex acts as a roadblock, sterically hindering the attachment of RNA Polymerase or preventing its progression along the DNA template [45]. This results in the potent and reversible silencing of the target gene without altering the underlying genetic code (Figure 1).

The accessibility of dCas9 is not uniform across the genome; it is heavily influenced by the physical state of the DNA and the surrounding cellular environment. For example, while euchromatin (active, loosely packed DNA) is accessible to dCas9, heterochromatin (regions of highly condensed DNA) can block the dCas9 from scanning the DNA for its PAM [46]. In cancer cells, certain tumor-suppressor genes may be hypermethylated, while oncogenes might be hypomethylated (highly accessible) [47]. Therefore, a CRISPRi may have different effects between a healthy cell and a diseased cell because the target site has been buried by epigenetic silencing.

Beyond dCas9, other Cas variants have expanded the CRISPRi toolkit. For example, dCas12a (formerly Cpf1) offers an alternative for AT-rich regions, utilizing a TTTN PAM and processing its own crRNA arrays, which facilitates multiplexed gene silencing [48,49].

### 2.2. Transcriptional Repression via Effector Domains

Steric hindrance achieved through dCas9 binding alone can repress transcription by physically obstructing the progression of RNA polymerase, but this effect is often modest and variable, typically resulting in a 60–80% knockdown of the target gene. For potent, near-complete gene silencing that persists over time, dCas9 is typically converted into a programmable scaffold by fusing it to one or more transcriptional repressor domains (Figure 1). The most widely utilized repressor domain is the Krüppel-associated box (KRAB) [50]. When the dCas9-KRAB fusion protein is recruited by the sgRNA to the promoter region of a target gene, the KRAB domain acts as a docking site for the co-repressor protein KAP1 (also known as TRIM28) [51]. KAP1, in turn, serves as a master scaffold, recruiting a suite of enzymatic machinery that induces localized, repressive epigenetic modifications. A critical component of this machinery is the heterochromatin protein 1 (HP1). The HP1 recognizes and binds methylated histone H3 lysine 9, produced by histone methyltransferase activity (SETDB1) which in turn recruits more HMTs, resulting in chromatin condensation of neighboring nucleosomes [52,53]. DNA methyltransferases (DNMTs) can also be fused with dCas9 to act as the gene silencer [54,55]. DNMTs can methylate cytosine residues in CpG islands, resulting in stable gene silencing. Such epigenetic editing transforms the accessible euchromatin surrounding the target promoter into inaccessible, condensed heterochromatin. This high-density chromatin architecture completely blocks the binding and progression of RNA Polymerase II and associated transcription factors, leading to the robust, heritable repression of gene expression at the transcriptional level. Histone deacetylases such as histone deacetylase 1 (HDAC1) can also be fused to dCas9 for gene silencing [56]. HDAC1 causes deacetylation of lysine residues on the N-terminal part of the core histones (H2A, H2B, H3 and H4) and histone deacetylation is a mark for epigenetic repression [57].

## 3. Targeting the Oncogenic Drivers with CRISPRi

The application of CRISPRi to inhibit oncogenic drivers represents a paradigm shift in precision oncology, offering a new strategy to silence previously undruggable targets. CRISPRi acts at the pre-transcriptional level, allowing for the potent suppression of diverse molecular classes, ranging from transcription factors to complex signaling kinases. Representative studies are summarized in Table 1.

### 3.1. Silencing Master Transcription Factors: MYC and Beyond

At the forefront of CRISPRi applications is the targeting of master regulators like MYC. As a “super-transcription factor” that lacks a traditional enzymatic pocket, MYC has historically been impossible to inhibit directly [58]. CRISPRi bypasses this by targeting the *MYC* promoter or its distal enhancers, effectively collapsing the transcriptional network that drives rapid cell proliferation and metabolic reprogramming in high-grade lymphomas and solid tumors [59,60,61]. Using a dCasX-KRAB CRISPRi delivered by Adeno-Associated Virus (AAV), Cao et al. successfully inhibited c-MYC and suppressed bladder cancer growth in vivo [62]. Similarly, other essential transcription factors that govern cancer stem cells or epithelial–mesenchymal transition (EMT), such as Oct4 and Nanog [63], can be potentially silenced by CRISPRi, stripping cancer cells of their invasive potential and resistance to chemotherapy.

### 3.2. Neutralizing Signaling Hubs: RAS and PI3K

The RAS and PI3K pathways are among the most frequently mutated signaling hubs in human cancer [64,65]. While specific inhibitors like covalent KRAS G12C blockers have seen clinical success [66], they are often met with primary and acquired resistance [67,68]. CRISPRi provides a more comprehensive solution by targeting the genomic source of these drivers. For example, a dCas9-HDAC1 fusion protein was used to silence KRAS in colon cancer [56]. Gao et al. employed adenovirus to deliver mutant KRAS-targeted CRISPRi to inhibit lung cancer growth in mice [69]. Based on these studies, we can predict that by silencing KRAS, NRAS, or the catalytic subunits of PI3K (PIK3CA), CRISPRi can be used to achieve a sustained reduction in downstream AKT and MAPK signaling. A significant advantage here is the ability to target the unique promoter sequences of mutated alleles or specific isoforms, potentially sparing wild-type signaling in healthy tissues. Such specificity is difficult to achieve with traditional kinase inhibitors.

### 3.3. Inhibition of Metastasis and Immunotherapy

CRISPRi is equally potent in disrupting the mechanisms that cancer cells use to metastasize. The HGF-PU.1-DPP4 axis can promote colorectal cancer metastasis to the liver. Wang et al. showed that CRISPRi silencing of DPP4 effectively suppressed liver metastasis in mice [70]. X-inactive specific transcript (XIST) is a long noncoding RNA (lncRNA). Cui et al. employed CRISPRi to silence XIST in breast cancer cells and inhibited tumor growth in mice and metastasis to the bone [71]. In the realm of immunotherapy, the CRISPRi technology can potentially be used to inhibit immune checkpoint proteins such as PD-1, PD-L1 and CTLA-4. These proteins are used by cancer cells to avoid attacks from the immune system [72]. The inhibition of one immune checkpoint (like PD-1) may not be enough, as other checkpoint proteins (Siglec-15, PD-L2, IDO1, etc.) can still suppress the immune cells. Since Cas12a can process its own CRISPR array to produce single crRNAs for multiplexed gene regulation [48], a dCas12a-based CRISPRi system can potentially be used to silence multiple immune checkpoints simultaneously.

**Table 1:** CRISPRi studies targeting oncogenes.

Gene Target	CRISPRi Format	Cancer Model	Delivery Strategy	Main Outcome	References
KRAS	dCas9-KRAB	Lung cancer	Adenovirus	Effective inhibition of tumor growth in xenograft model	[69]
KRAS	dCas9-HDAC1	Colon and pancreatic cancers	Plasmid or recombinant protein	Inhibition of tumor growth in vitro	[56]
ΔNp63	dCas9-KRAB	Squamous cell carcinomas (SCCs)	Adenovirus	Effective inhibition of tumor growth in xenograft model	[73]
MYC	dCas9-KRAB	Acute Myeloid Leukemia (AML)	Lentivirus	Inhibition of cancer cell viability in vitro	[74]
MYC	dCasX-KRAB	Bladder cancer	Adeno-associated Virus (AAV)	Effective inhibition of tumor growth in xenograft model	[62]
DPP4	dCas9-KRAB	Colon cancer	Transfection	Effective inhibition of tumor growth and metastasis in vivo	[70]
XIST	dCas9-KRAB	Breast cancer	Transfection	Effective inhibition of tumor growth and metastasis in vivo	[71]

## 4. Advantages of CRISPRi over Current Gene Silencing Technologies

The fundamental difference in the mechanism of action—targeting DNA versus mRNA—bestows CRISPRi with significant advantages for therapeutic oncogene silencing. By arresting gene expression at the genomic source, CRISPRi provides more robust and persistent gene repression than technologies that rely on the continuous degradation of rapidly regenerating mRNAs. Furthermore, this DNA-level intervention avoids the common off-target toxicities associated with the RNAi machinery, offering a highly programmable and specific platform for blocking the undruggable oncogenes.

### 4.1. Improved Specificity and Reduced Off-Target Effects

CRISPRi exhibits a substantially reduced propensity for off-target silencing compared to the inherent limitations of RNAi, primarily due to the disparate stringency of their respective binding mechanisms. While RNAi relies on the incorporation of siRNA fragments into the endogenous RISC, its specificity is often compromised because the 6–7 nucleotides long seed region can tolerate multiple mismatches [75,76,77]. This lack of fidelity frequently leads to the unintended degradation of non-target mRNAs with partial sequence complementarity, a phenomenon that translates into significant cellular toxicity and adverse events in clinical settings.

In contrast, the dCas9-sgRNA complex provides a high level of targeting precision by requiring both a 20-nucleotide guide sequence and the presence of a specific PAM for stable DNA binding [78]. The dCas9 protein is highly sensitive to mismatches, particularly within the PAM-proximal seed region, ensuring that the transcriptional roadblock is only established at the intended genomic locus [79]. By shifting the therapeutic target from the volatile and numerous mRNA transcripts to a single genomic source, CRISPRi avoids the widespread off-target signature of RNAi, offering a safer and more predictable platform for the systemic silencing of oncogenic drivers.

The off-target effects do occur when dCas9 acts on untargeted genomic sites [80]. This problem can be addressed with several strategies. High-fidelity variant of dCas9 can be used to achieve higher on-target activity [81]. New Cas9 homologs that use rarer PAM sequences (such as St1Cas9 and St3Cas9) can be used which is less likely to bind to the non-targeted DNA region [82]. The sequence of sgRNAs can affect the off-target effects. Different sgRNAs targeting the same gene can have distinct outcomes, thus it is important to screen for the best performing sgRNA for the target gene.

### 4.2. Stable and Long-Term Gene Repression

The fundamental distinction between CRISPRi and RNAi lies in the durability of their silencing effects, a factor that is particularly critical for the long-term management of chronic conditions such as cancer. RNAi-mediated knockdown is inherently transient because it targets the volatile pool of mRNA; as siRNAs are degraded by cellular nucleases, the continuous production of new mRNAs necessitates repetitive, high-dose administrations to maintain therapeutic efficacy. This cycle of frequent dosing not only complicates clinical protocols but also significantly escalates the risk of immunogenicity and cumulative off-target toxicities. Recent advances in delivery technology have improved the effectiveness of siRNA therapy, resulting in FDA approval of drugs like inclisiran, which can be given twice per year to inhibit Proprotein Convertase Subtilisin/Kexin Type 9 (PCSK9) in the liver to reduce the risk of developing atherosclerotic cardiovascular disease [83].

In contrast, CRISPRi offers a long-acting alternative by operating at the epigenetic level. When dCas9 is fused to a repressor like the KRAB domain, it initiates a cascade of chromatin compaction and histone modifications that can be maintained through multiple rounds of cell division. This epigenetic memory allows for a single therapeutic intervention to provide prolonged gene inhibition at the genomic source, rather than chasing a regenerating population of mRNAs. In fact, a recent study demonstrated that a single dose of the lipid nanoparticle (LNP)-encapsulated CRISPRi was sufficient to silence the PCSK9 gene in mice for at least one year [84]. Consequently, CRISPRi’s DNA-level action enables a more practical and robust clinical application, achieving sustained oncogene suppression with a significantly reduced dosing frequency compared to post-transcriptional silencing methods.

### 4.3. Targeting Non-Coding Oncogenic Elements

CRISPRi’s capacity to target any sequence on the DNA provides unparalleled access to regulatory elements, a feat that RNAi (which is restricted to the coding sequence of processed mRNA) cannot achieve. By directing the dCas9 complex to an oncogene’s promoter, the specific binding sites of master transcription factors, or even distant enhancer elements, researchers can effectively collapse the transcriptional architecture of a target gene without needing to target the coding sequence itself.

This regulatory flexibility also enables precise gene family differentiation; because CRISPRi can be programmed to target unique, non-coding regulatory regions, it allows for the selective silencing of a single isoform or a specific member of a gene family, such as individual RAS or MYC isoforms. This level of precision is often impossible for sequence-homology-dependent RNAi or ASOs, which frequently struggle to distinguish between transcripts that share high sequence identity within their coding regions. By exploiting the unique genomic fingerprint of a gene’s control regions, CRISPRi offers a more sophisticated and specific approach to neutralizing the drivers of tumor progression.

### 4.4. Reversible Knockdown

Unlike the irreversible gene knockouts (KO) created by traditional CRISPR-Cas9, which rely on the induction of DSBs, CRISPRi achieves a non-mutagenic knockdown (KD) that significantly enhances the safety profile of therapeutic gene silencing. By avoiding DSBs entirely, CRISPRi eliminates the risks associated with error-prone DNA repair pathways, which can lead to unintended genomic deletions, translocations, or chromosomal instability.

This approach also offers tunable gene repression which is vital for targeting essential genes that are required for general cell survival but are overexploited by cancer cells; a complete knockout might be lethal to healthy tissue, whereas a calibrated knockdown can specifically neutralize the oncogene addiction of the tumor. By modulating the expression levels of the dCas9-KRAB complex, CRISPRi can effectively mimic the kinetics of a pharmacological inhibitor, providing a flexible and safer alternative for precision oncology. For example, a molecular glue degradation system was designed to degrade Cas9 in the presence of the FDA-approved drug, pomalidomide [85]. Similarly, a chemogenetic system was developed to bring Cas9 to a ubiquitin ligase, enabling rapid ubiquitination and degradation of Cas9 by the proteasome [86]. These methods offer a control mechanism to turn off the CRISPRi machinery if it is needed to reduce the toxicity.

## 5. Delivery Systems for CRISPRi

The delivery of CRISPRi is the main challenge in clinical translation. While the molecular machinery of dCas9-KRAB is highly effective at silencing oncogenes, transporting these large components into the tumor cells remains a pressing obstacle. Current strategies to overcome the delivery barrier include viral vectors, non-viral systems, and sophisticated tumor-targeting modalities.

### 5.1. Viral Delivery Systems

Viral vectors exploit the natural ability of viruses to infect cells and deposit genetic material. Among these, lentiviral vectors are excellent for in vitro and ex vivo applications due to their high transduction efficiency and ability to integrate into the host genome, ensuring permanent expression of the CRISPRi machinery [87]. However, for systemic cancer therapy, the risk of insertional mutagenesis and the host’s immune response limit their in vivo utility. AAV vectors are the preferred vehicle for in vivo delivery due to their low immunogenicity and diverse tissue tropism [88]. Several labs have successfully developed AAV-based delivery of CRISPRi to silence genes in vitro and in vivo. For example, Backstrom et al. optimized CRISPRi components to generate a single AAV vector that effectively knocks down PCSK9 expression in vivo [89]. Laurette et al. developed an AAV-based method for CRISPRi-mediated epigenetic silencing of gene expression in cardiac myocytes in vitro and in vivo [90].

### 5.2. Non-Viral Delivery: LNPs and Exosomes

**Figure 2:**
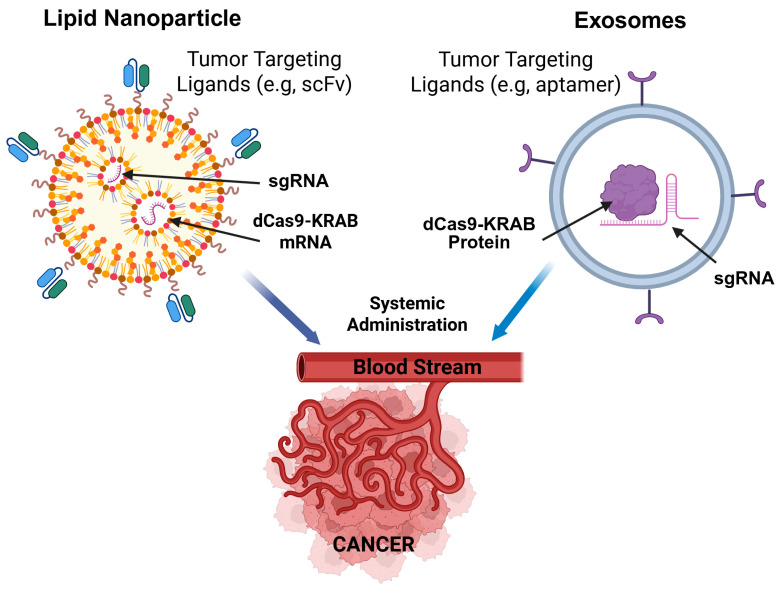
Targeted delivery of CRISPRi for cancer therapy. Lipid nanoparticles can be decorated with tumor-targeting ligands (such as scFv for EGFR) to deliver the CRISPRi components (dCas9-KRAB mRNA and sgRNA) to the cancer cells. The exosomes can be loaded with dCas9-KRAB protein and sgRNA RNP complex. Targeting ligands (such as aptamers) can be used to guide the exosomes to cancer cells. Both LNPs and exosomes can be administered intravenously to reach primary tumors as well as the distant metastasis. Created in BioRender. Guo, B. (2026) https://BioRender.com/r83soua.

Non-viral systems offer a safer, more flexible alternative approach to avoid the risks of permanent genomic integration and long-term immunogenicity. Lipid Nanoparticles (LNPs) have emerged as the frontrunner, following their global success in mRNA vaccines [91]. LNPs can encapsulate the large dCas9 mRNA and the small sgRNA within an ionizable lipid shell (Figure 2). Once they reach the tumor cell, they are internalized via endocytosis and undergo endosomal escape, releasing the mRNA into the cytoplasm for translation [92]. This transient expression is ideal for cancer therapy, as it provides a therapeutic window of gene silencing without the toxic accumulation of dCas9 protein. Furthermore, exosomes (natural extracellular vesicles) are being explored as an effective delivery system for nucleic acids and proteins [93]. Because exosomes are cell-derived, they exhibit superior biocompatibility and can bypass certain immune clearance mechanisms that often trap synthetic nanoparticles [94]. In addition, exosomes can be engineered with RNA nanoparticle decoration to avoid endosome trapping [95]. CRISPRi components can be loaded into the engineered exosomes for targeted delivery to the cancer cells (Figure 2).

### 5.3. Advanced Tumor-Targeting and Triggered Release

To minimize systemic side effects, delivery vehicles are increasingly armed with tumor-targeting strategies. By decorating the surface of LNPs or exosomes with ligands such as monoclonal antibody, single-chain variable fragment (scFv), peptide, aptamer, or small molecules like folate, tumor-specific delivery (Figure 2) can be achieved when the ligands specifically bind to receptors overexpressed on cancer cells (e.g., EGFR or folate receptor) [96]. Beyond surface targeting, smart delivery systems are engineered to respond to the unique tumor microenvironment (TME). pH-responsive systems can exploit the acidic nature of the TME to trigger the release of the dCas9 cargo only at the tumor site [97]. Similarly, enzymatically cleavable linkers can be used to shield the nanoparticle during circulation, only revealing its active binding motifs when they encounter specific proteases (like MMPs) secreted by invasive cancer cells [98]. These multi-layered delivery approaches will transform CRISPRi from a laboratory tool into a viable, precision-guided therapeutic.

## 6. Translational Challenges and Future Directions

Despite its potential, the clinical translation of CRISPRi for systemic cancer therapy faces significant, shared challenges with other nucleic acid-based therapeutics, primarily revolving around delivery, specificity, and immunogenicity.

### 6.1. The Delivery Bottleneck

Translating CRISPRi into a viable clinical therapy hinges on the efficient co-delivery of its two primary molecular components (the dCas9-KRAB fusion and the sgRNA) to cancer cells in vivo. AAVs have served as the predominant delivery vehicle; however, the dCas9 cassette frequently exceeds the strict 4.7 kb packaging limit of a single AAV. This constraint necessitates complex engineering solutions, such as utilizing miniaturized Cas orthologs or optimizing the promoter length that drives dCas9 expression. Consequently, non-viral platforms like LNPs emerge as versatile alternatives. LNPs can encapsulate dCas9 mRNA and sgRNA, avoiding the risks of genomic integration. Despite this potential, achieving high-level accumulation within the dense stroma of solid tumors and ensuring subsequent endosomal escape remain significant physiological hurdles [92,99]. To overcome these barriers and mitigate systemic toxicity, current research focuses on decorating LNPs with tumor-targeting ligands or cell-penetrating peptides. RNA nanotechnology has been used to construct folate-displaying exosomes which can deliver the payloads while avoiding endosome trapping [100]. These modifications are essential to concentrate the CRISPRi machinery within the tumor microenvironment, ensuring potent epigenetic silencing while sparing healthy tissues from off-target effects.

### 6.2. Immunogenicity

As a bacterial protein derived from S. pyogenes, dCas9 is a foreign antigen that can trigger potent immune responses in humans. Prior exposure to common bacteria or repeated therapeutic delivery can elicit neutralizing antibodies and inflammation, compromising both safety and efficacy [101]. To mitigate this immunogenicity, researchers can employ non-immunogenic LNPs. By encapsulating dCas9 mRNA within LNPs, the protein is expressed only transiently, minimizing its exposure to the immune system compared to the persistent expression seen with viral vectors.

### 6.3. Regulatory and Ethical Considerations

While CRISPRi avoids the permanent, irreversible genomic alterations associated with nuclease-based Cas9, its capacity for sustained epigenetic repression necessitates a rigorous assessment of long-term safety. Unlike a transient siRNA knockdown, the dCas9-KRAB-induced heterochromatin can persist through multiple cell divisions, raising critical questions regarding the potential for unintended, chronic silencing in healthy tissues. Because these epigenetic modifications can be remarkably stable, the precise window of reversibility must be characterized to ensure that gene function can be restored if toxicity occurs. Future clinical applications will likely require inducible control systems or self-inactivating circuits to balance the potency of long-term oncogene suppression with the necessity of a safety off switch to protect the patients.

## 7. Conclusions

CRISPRi is a new gene silencing technology, offering precision and transcriptional repression stability. By targeting oncogene expression directly at the DNA level, CRISPRi provides a potent platform for tackling the vast landscape of cancer, especially those driven by undruggable oncogenes and transcription factors. While challenges in in vivo delivery and immunogenicity persist, the rapid advancements in vector engineering and nanotechnology discovery will move CRISPRi-based therapeutics from the preclinical stage to clinical trials.

## Data Availability

No new data were created or analyzed in this study.

## Abbreviations

The following abbreviations are used in this manuscript:

CRISPRi	CRISPR interference
CRISPR	Clustered regularly interspaced palindromic repeats
RNAi	RNA interference
ASO	antisense oligonucleotides
KRAS	Kirsten rat sarcoma gene
EGFR	Epidermal Growth Factor Receptor
siRNA	small interfering RNA
shRNA	short hairpin RNA
miRNA	microRNA
dCas9	catalytically dead Cas9
RNP	Ribonucleoprotein
PAM	Protospacer Adjacent Motif
KRAB	Krüppel-associated box
HP1	heterochromatin protein 1
DNMT	DNA methyltransferase
HDAC1	Histone Deacetylase 1
EMT	epithelial–mesenchymal transition
PI3K	Phosphoinositide 3-kinase
PD-1	Programmed Cell Death 1
PD-L1	Programmed death-ligand 1
RISC	RNA-induced Silencing Complex
LNP	lipid nanoparticle
DSB	double-strand break
scFv	single-chain variable fragment
MMPs	Matrix Metalloproteinases
TME	tumor microenvironment
AAV	Adeno-associated virus
